# Vascular Endothelial Growth Factor, Irradiation, and Axitinib Have Diverse Effects on Motility and Proliferation of Glioblastoma Multiforme Cells

**DOI:** 10.3389/fonc.2017.00182

**Published:** 2017-08-22

**Authors:** Reinhardt Krcek, Veronika Matschke, Verena Theis, Irenäus Anton Adamietz, Helmut Bühler, Carsten Theiss

**Affiliations:** ^1^Department of Cytology, Institute of Anatomy, Ruhr-University Bochum, Bochum, Germany; ^2^Department of Radiotherapy and Radio-Oncology, University Medical Centre Marien Hospital, Ruhr-University Bochum, Herne, Germany; ^3^Institute for Molecular Oncology, Radio-Biology and Experimental Radiotherapy, University Medical Centre Marien Hospital, Ruhr-University Bochum, Herne, Germany

**Keywords:** glioma, cell motility, cell proliferation, vascular endothelial growth factor, irradiation, axitinib, videography

## Abstract

Glioblastoma multiforme (GBM) is the most common primary brain tumor. It is highly aggressive with an unfavorable prognosis for the patients despite therapies including surgery, irradiation, and chemotherapy. One important characteristic of highly vascularized GBM is the strong expression of vascular endothelial growth factor (VEGF). VEGF has become a new target in the treatment of GBM, and targeted therapies such as the VEGF-receptor blocker axitinib are in clinical trials. Most studies focus on VEGF-induced angiogenesis, but only very few investigations analyze autocrine or paracrine effects of VEGF on the tumor cells. In this study, we examined the impact of VEGF, irradiation, and axitinib on cell proliferation and cell motility in human GBM cell lines U-251 and U-373. VEGF receptor 2 was shown to be expressed within both cell lines by using PCR and immunochemistry. Moreover, we performed 24-h videography to analyze motility, and a viability assay for cell proliferation. We observed increasing effects of VEGF and irradiation on cell motility in both cell lines, as well as strong inhibiting effects on cellular motility by VEGF-receptor blockade using axitinib. Moreover, axitinib diminished irradiation induced accelerating effects. While VEGF stimulation or irradiation did not affect cell proliferation, axitinib significantly decreased cell proliferation in both cell lines. Therefore, the impairment of VEGF signaling might have a crucial role in the treatment of GBM.

## Introduction

Glioblastoma multiforme (GBM) is the most common primary brain tumor in adults and is highly malignant. Its etiology is widely unknown with an incidence of 3–4/100,000 and a poor outcome for the patients. The tumor is preferentially located in the frontal lobes of the supratentorial compartments, but can also occur focally, multifocally or diffusely in all cortical areas (1, 2). The current standard therapy is a combination of surgery, a fractionated radiotherapy and a chemotherapeutic treatment with DNA alkylating temozolomide. However, it offers only a median survival time of 15 months (2). One reason for this poor prognosis is the high invasiveness of GBM into the brain parenchyma, hindering a complete resection in most cases (3–5). Despite of an improvement using radiotherapy to reduce the tumor mass, it is not possible to achieve high survival rates (6).

As GBM shows high levels of vascularization (1), one new target is the vascular endothelial growth factor (VEGF) which plays an important role, especially in angiogenesis (4, 7). VEGF was first described in 1983 as a protein in the tumor ascites fluids of guinea pigs, where it promoted vascular permeability (8). It is a dimeric polypeptide and constitutes a gene family including VEGF-A, VEGF-B, VEGF-C, VEGF-D, and placental growth factor. VEGF-A, in the following referred to as VEGF, is the most important and best known form of VEGF, and is one of the key regulators in angiogenesis, stimulating proliferation, chemotaxis, survival, and permeability of endothelial cells (7, 9–11). VEGF is the activating ligand of two receptor-tyrosine kinases, VEGF receptor 1 (VEGF-R1) and VEGF receptor 2 (VEGF-R2). While VEGF-R1 is supposed to play a role mainly as a modulator of VEGF-R2-mediated signaling (12), the main effects in angiogenesis occur *via* activation of VEGF-R2, for instance through activation of Cdc42 and SAPK2/p38, which leads to a remodeling of actin (13).

In the brain, VEGF is mainly expressed by neurons, astrocytes, and endothelial cells (14). Some stimuli for the release of VEGF are known as hypoxia inducible factors, which are activated by insufficient blood supply, for instance in fast growing cancer (15). Another mechanism of VEGF secretion is mediated by stimulation of MAPK dependent pathways, for example because of irradiation (16).

It is well described that several tumors like hepatocellular cancer or the highly vascularized GBM produce high levels of VEGF to force neoangiogenesis and growth (17–19). Therefore, VEGF became a target in the treatment of cancer. One example is bevacizumab, the first humanized antibody against VEGF, which has been proved to be a successful supplementation to standard therapies in colorectal cancer (20, 21). More recently, several clinical trials are testing the efficiency of bevacizumab in high-grade glioma, offering encouraging results in terms of an increase in the progression free survival period (22, 23). Another candidate for anti-angiogenic therapy is the tyrosine kinase inhibitor axitinib, a selective inhibitor of the VEGF receptors 1, 2, and 3, passing through phase III of clinical testing in renal carcinoma (24, 25) and in phase II for GBM (NCT01562197). Besides stimulating angiogenesis, VEGF also shows proliferative effects on several tumors (26). Furthermore, it has been shown that VEGF enhances proliferation and motility in glial cells (27). Based on these data derived from cultured astrocytes, we analyzed the impact of VEGF, irradiation and axitinib on proliferation and motility in the two different human GBM cell lines U-251 and U-373.

## Materials and Methods

### Materials

VEGF-A (Sigma-Aldrich, V4512, USA) and axitinib (Selleckchem, S1005, USA) was added to cell culture medium in a concentration of 0.1 and 10 µg/ml, respectively. To investigate the impact of therapeutic irradiation, cells were irradiated with the linear accelerator, Elekta Synergy S, at 5 Gy/min at the university clinic Marien Hospital Herne (Germany). Cell medium was changed 1 h after irradiation.

### Cell Lines

Two human glioblastoma cell lines were used. U-251 MG human brain glioblastoma cell line was obtained from CLS (Heidelberg, Germany) and U-373 MG was a generous gift from Dr. Bardenheuer (Essen, Germany). The cell lines U-251 and U-373 were routinely grown in Dulbecco’s modified eagle medium (DMEM) with 4.5 g/l d-Glucose, 3.7 g/l NaHCO_3_, stable glutamine and Na-pyruvat (Biochrom AG, FG 0445, Germany). Media were supplemented with 10% sterile fetal bovine serum (Biochrom AG, S 0115) and 1% penicillin–streptomycin (Sigma-Aldrich, P0781). Cell lines were maintained at 37°C, 5% CO_2_, and 90% humidity.

### Immunohistochemistry

Experiments started 1 day after seeding of the GBM cell lines at a density of 5,000 cells/12 mm cover slip. The cells were fixed with 4% paraformaldehyde for 10 min, followed by permeabilization with 0.1% Tween (VWR, 663684B, USA) in PBS for 60 min. Additionally, unspecific binding sites were blocked with goat-serum (Sigma-Aldrich, G9023, 10% in PBS) and bovine serum (Sigma-Aldrich, B9433, 3% in PBS) adding 0.3 M glycine (Biomol, 04943, Germany). After washing with PBS, cells were incubated with rabbit anti-VEGF-R2 antibody overnight (Abcam, ab39638, United Kingdom, 1:300 in PBS), followed by incubation with AlexaFluor 488 anti-rabbit IgG (Molecular Probes, A-11008, USA, 1:250 in PBS) for 75 min and then subsequently treated with rhodamine-phalloidin for 30 min (Sigma-Aldrich, P1951, 1:20 in PBS). Further bisBenzimide H 33342 trihydrochloride (Hoechst, Sigma-Aldrich, B2261, 1:1,000 in PBS, 20 min) was applied to counterstain the cell nuclei. Finally, the cover slips were mounted on microscope slides with fluoromount mounting medium (Dako, S3023, Germany).

### Reverse Transcription-PCR

To prove the existence of VEGF-R1 (*FLT1*) und VEGF-R2 (*KDR*) mRNA in GBM cell lines a qualitative PCR was performed. Total RNA was isolated using ReliaPrep RNA Cell Miniprep System (Promega, Z6011, USA) according to manufacturer’s protocol. cDNA was synthesized from 1 µg of total RNA using Promega Reverse Transcription System (Promega, Z6011, USA). We used prior published specific primer sequences for amplification of the gene of interest: *FLT1*—forward: ATCATTCCGAAGCAAGGTGTG, reverse: AAACCCATTTGGCACATCTGT, *KDR*—forward: AGGCAGCTCACAGTCCTAGAGC, reverse: GTCTTTTCCTGGGCACCTTCTA (28). PCR was performed using the GoTaq G2 Hot Start Green Master mix (Promega, M7422). PCR products were analyzed by agarose gel electrophoresis before verification by sequencing.

### Videography

Time-laps videography was performed as described previously (29). In brief, after seeding 50,000 cells/3.5-cm well, cells were treated with VEGF (0.1 µg/ml), axitinib (10 µg/ml), and/or radiation (2 Gy) on the next day. Medium was changed 1 h after irradiation containing the reagents. At the same time, the medium of non-irradiated glioma cells was changed. Videography started 2 h after adding fresh medium.

Cells were maintained in a 5% CO_2_ atmosphere at 37°C and observed for 24 h, taking images every 15 min using an inverse microscope (Zeiss Axiovert 25) equipped with a 10× objective (Zeiss A-plan 10, NA 0.25) and an Olympus E420 camera. For analysis of cellular motility, cells were tracked throughout the image stack and then analyzed by the Chemotaxis and Migration tool V2.0 (ibidi^®^). All cells that stayed in focus for at least 5 h were examined. The measured velocity of each cell was normalized to the average of the control conditions and plotted into a bar chart.

### Proliferation Assay

5,000 cells per well were seeded in a 96-well plate (Sarstaedt, Germany). On the next day, cells were treated with irradiation, VEGF, and/or axitinib. Fresh medium was added 1 h after irradiation including the reagents, with the same procedure for non-irradiated cells. 24 h after treatment, cells were incubated with MTS reagents (Promega CellTiter 96^®^ AQueous Non-Radioactive Cell Proliferation Assay, G5421) for 2 h. Thereafter, absorbance at 450 nm was measured with a Multiscan Ascent 354 (Labsystems, Heidelberg, Germany). The measured values were normalized to the average of the control conditions and plotted into a bar chart.

### VEGF ELISA—Analysis of Supernatant

One million cells were seeded in a 6-cm petri-dishe (Sarstedt, Germany). After 24 h the medium was replaced by 4 ml DMEM containing 1% fetal bovine serum. Subsequently both cell lines were irradiated with 2 Gy. On the next day, the supernatant was removed, diluted 1:5 in DMEM containing 1% fetal bovine serum, and the VEGF ELISA was performed as described in the manufacturer’s protocol (Human VEGF Quantikine ELISA Kit, Bio-Techne, DVE00, Wiesbaden, Germany).

### Statistical Analysis

Statistical analyses of the data were performed with Prism 5.0 (Graph Pad Inc., La Jolla, CA, USA). Data represent mean values of at least three independent experiments ± SE of the mean (SEM). Experiments were analyzed by one-way ANOVA with Bonferroni multiple comparison post-test. *p*-Values *p* ≤ 0.05 were considered statistically significant.

## Results

### VEGF Receptor Expression

**Figure 1:**
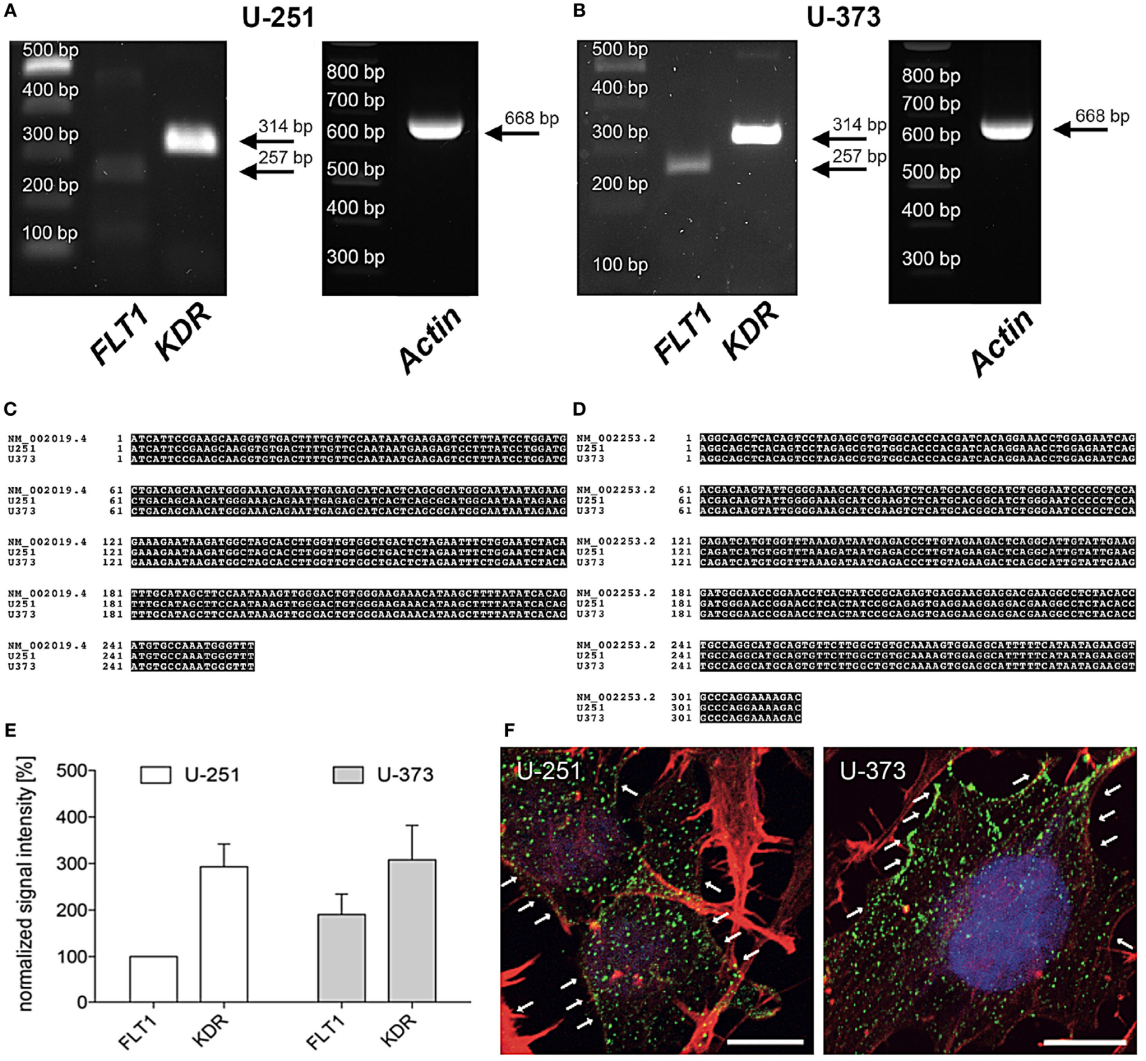
Expression of vascular endothelial growth factor (VEGF)-R2 (*KDR*) in U-251 and U-373 glioblastoma cell lines. **(A,B)** VEGF-R1 (*FLT*1) as well as VEGF-R2 (*KDR)* are expressed in both cell lines U-251 **(A)** and U-373 **(B)**. β*-Actin* was used as housekeeping gene. PCR products were isolated and confirmed by DNA sequencing. **(C,D)** Sequence of amplified PCR product for *FLT1* **(C)** and *KDR* **(D)** matches their specific database entries (*FLT1* – NM_002019.4; *KDR* – NM_002253.2) and were proven to be unique in human glioblastoma cell lines U-251 as well as in U-373 by comparison with a database (Blast 2.2, U.S. National Centre for Biotechnology Information, Bethesda, MD, USA) **(E)**. Semi-quantitative analysis of gene expression normalized to β*-Actin* and compared to the expression of U-251-FLT (100%). Data are shown as mean ± SEM. *n* = 3. **(F)** Both glioblastoma multiforme cell lines express VEGF-R2 (green dots, arrows) in the cytoplasm as well as along the cell membrane. Counterstaining of the actin cytoskeleton is given in red as well as cell nuclei staining with DAPI in blue. Scale bars: 10 µm.

In line with former studies the expression of VEGF-R2 (*KDR*) mRNA was verified by PCR and immunohistochemistry (30, 31). Figure 1 shows that the human glioblastoma cell lines U-251 and U-373 both express VEGF-R1 (*FLT1*) and VEGF-R2 (*KDR*) (A, B). DNA sequencing of the PCR products confirmed these results (C, D). Moreover, VEGF-R2 (*KDR*) is much stronger expressed than VEGF-R1 (*FLT1*) in both cell lines (E). In addition, the distribution of VEGF-R2 was detected immunohistochemically within the cytoplasm as well as along the cell membrane in both GBM cell lines (F).

### Quantitative Analysis of Cellular Motility

**Figure 2:**
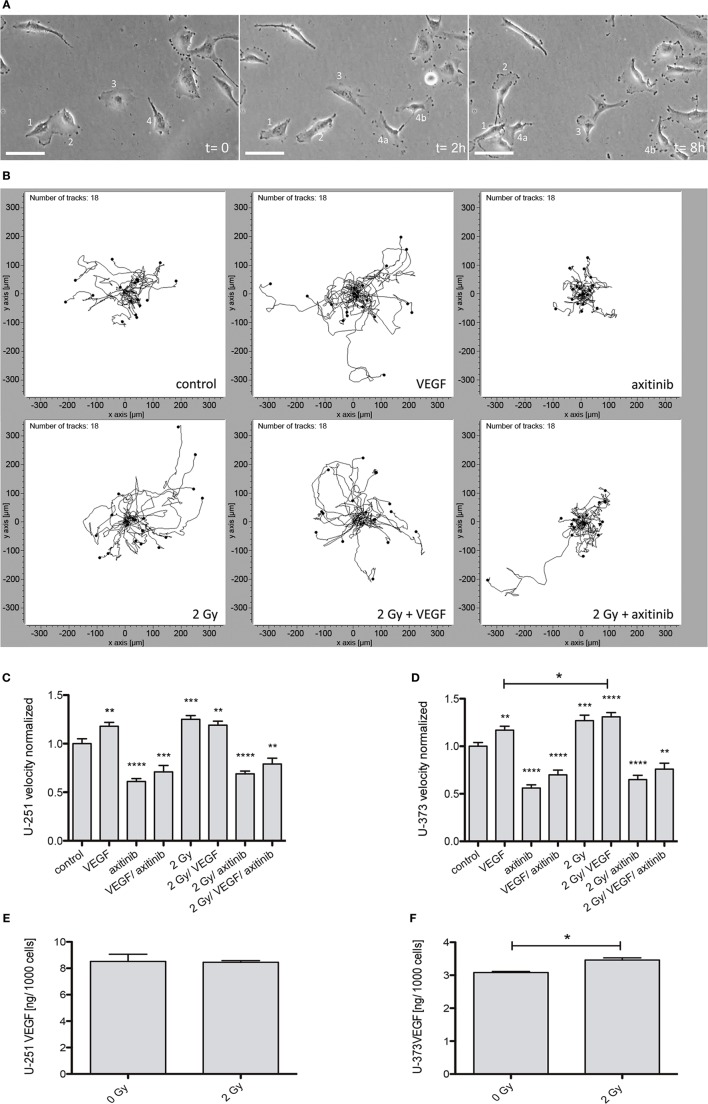
Influence of vascular endothelial growth factor (VEGF), axitinib, or irradiation with 2 Gy photons on the motility of glioblastoma multiforme cell lines U-251 and U-373. The motility of the cells was analyzed by time-laps videography. Cells were tracked and analyzed with the ibidi chemotaxis and migration tool. **(A)** Examples from an image stack of Videography. Some cells migrate fast (2), some slowly (3), some even do not migrate (1). Dividing cells (4) often keep contact over longer periods of time (4a, 4b). Scale bars: 50 µm. **(B)** Migration of U-373 glioblastoma cells under varied conditions. Depicted are the tracked cells in representative fields of view. The software merges all starting points in the origin to get an explicit view of the paths migrated by the cells. It is clearly visible that the migration is undirected (an advantage of videography over other methods to analyze migration). It is notable that some irradiated cells are able to escape the inhibition by axitinib. **(C,D)** The motility of U-251 and U-373 cells is increased by VEGF as well as by irradiation. In U-373, a combination of both leads to a significant increase in velocity compared to VEGF alone **(D)**, in U-251 no additive effects could be observed. In contrast, axitinib diminishes the motility of untreated cells and the elevated motility after irradiation as well. VEGF and axitinib were added in concentrations of 0.1 and 10 µg/ml, respectively. **(E,F)** 24 h after 2 Gy irradiation the amount of VEGF in the supernatant of U-251 and U-373 was analyzed. In U-251 there were no significant changes detectable, whereas in U-373 cells VEGF was significantly increased **(F)**. Data are shown as mean ± SEM. Data were tested for significance using one-way ANOVA with Bonferroni multiple comparison post-test. Significant differences are indicated by *p < 0.05; **p < 0.01; ***p < 0.001; ****p < 0.0001.

To determine the effects of VEGF, irradiation, or axitinib treatment on cell motility, 24 h videography was performed (Figure 2). A set of samples of time-lapse videography of U-373 cells is shown in Figure 2A as well as a representative plot of tracked cells Figure 2B. All values being compared to untreated controls were presented for both cell lines: U-251 (Figure 2C) and U-373 (Figure 2D). Adding VEGF significantly enhanced the velocity of cellular motility in U-251 (from 1.0 ± 0.051 untreated to 1.18 ± 0.039, *n* = 60, *p* < 0.001), and in U-373 (from 1.0 ± 0.039 untreated to 1.17 ± 0.041, *n* = 60, *p* < 0.01). Irradiation also showed significantly increasing effects in U-251 (1.25 ± 0.04, *n* = 60, *p* < 0.001) and in U-373 cells (1.27 ± 0.057, *n* = 60, *p* < 0.001). However, in U-251 cells the combination of adding VEGF plus irradiation does not lead to higher levels of cellular motility, compared with cells only treated with VEGF or 2 Gy (1.19 ± 0.042). In U-373 the combined treatment with VEGF and 2 Gy had a significant effect on cell velocity, compared with VEGF alone (1.31 ± 0.045, *n* = 60, *p*(VEGF) < 0.05). Axitinib treatment had pronounced and significant decelerating effects on both cell lines, U-251 (0.61 ± 0.031, *n* = 60, *p* < 0.0001), and U-373 (0.56 ± 0.034, *n* = 60, *p* < 0.0001). The combinations of VEGF and axitinib (U-251: 0.71 ± 0.066, *n* = 60, *p* < 0.001; U-373: 0.70 ± 0.049, *n* = 60, *p* < 0.0001) or irradiation with 2 Gy plus axitinib treatment showed similar effects as with axitinib alone in U-251 (0.69 ± 0.028, *n* = 60, *p* < 0.0001), and in U-373 (0.65 ± 0.044, *n* = 60, *p* < 0.001). The partly pronounced effects of irradiation plus axitinib were not significant with respect to axitinib alone. The combination of all three treatments, irradiation (2 Gy), VEGF, and axtinib exhibited a significant decrease in cellular motility compared to control in both cell lines (U-251: 0.79 ± 0.06, *n* = 60, *p* < 0.001; U-373: 0.76 ± 0.062, *n* = 60, *p* < 0.001).

### Analysis of VEGF Levels in the Supernatant in U-251 and U-373

Additionally, we analyzed the amount of VEGF within the supernatant of U-251 and U-373 24 h after 2 Gy irradiation (Figures 2E,F). In at least six independent experiments for each cell line the supernatant of U-373 (Figure 2F) revealed a significant increase of VEGF (3.461 ± 0.068 vs. untreated 3.083 ± 0.032 pg per 1,000 cells, *n* = 6, *p* < 0.05), whereas in U-251 (Figure 2E) the amount of VEGF in the supernatant did not show any significant changes after irradiation.

### Impact of VEGF and Irradiation on Cell Proliferation

**Figure 3:**
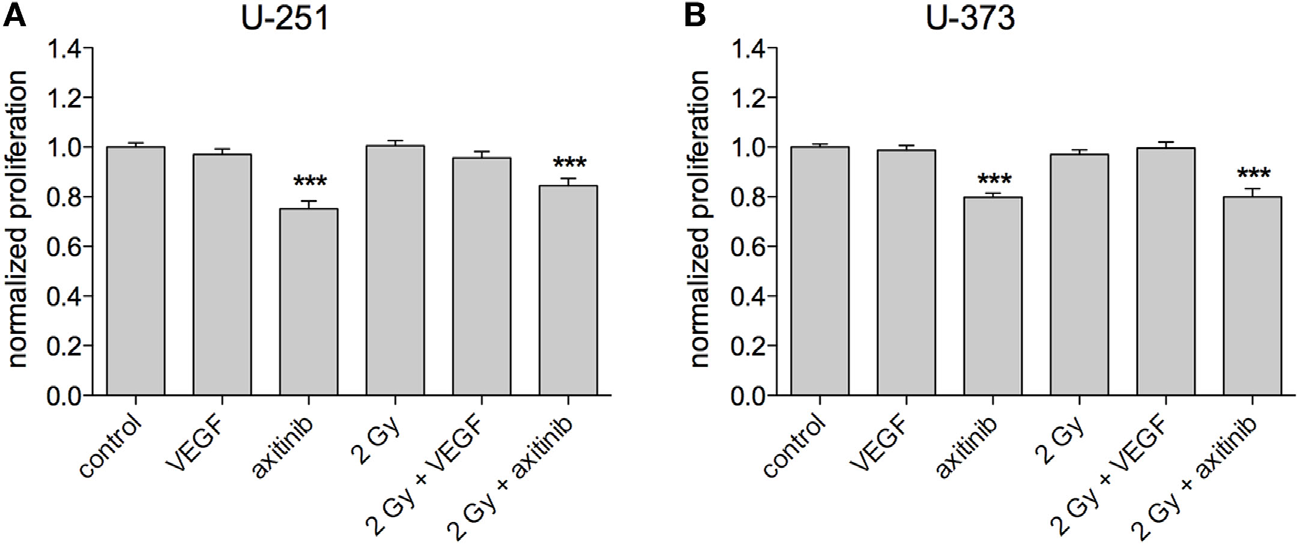
Influence of vascular endothelial growth factor (VEGF), axitinib, or irradiation with 2 Gy photons on the proliferation of glioblastoma multiforme cell lines U-251 and U-373. **(A,B)** Axitinib impairs the proliferation of irradiated and non-irradiated U-251 and U-373 cells. VEGF, irradiation, or the combination of both has no significant effect on cell proliferation. The proliferation of the cells was analyzed by a modified MTS-test. VEGF and axitinib were added in concentrations of 0.1 and 10 µg/ml, respectively. Data are shown as mean ± SEM. Data were tested for significance using one-way ANOVA with Bonferroni multiple comparison post-test. Significant differences are indicated by ***p < 0.001.

To investigate the influence of treatment on GBM proliferation, the Promega MTS assay was used, with at least three independent experiments for each cell line (Figures 3A,B). VEGF, irradiation with 2 Gy, or a combination of irradiation and VEGF had no significant effects on proliferation in all tested cell lines. However, the VEGF receptor blocker axitinib decreased the proliferation of U-251 (0.752 ± 0.032, *n* = 19, *p* < 0.001), and of U-373 (0.798 ± 0.017, *n* = 20, *p* < 0.001). Similar results were obtained for the combination of axitinib and irradiation in U-251 (0.844 ± 0.029, *n* = 25, *p* < 0.001), and in U-373 (0.8 ± 0.032, *n* = 20, *p* < 0.001). In U-251, there was a significant increase in proliferation comparing axitinib to irradiation plus axitinib (0.752 ± 0.032 vs. 0.844 ± 0.029, *p* < 0.05), whereas U-373 showed no significant difference between these two treatments.

## Discussion

High-grade glioma patients have a poor prognosis despite all the options of today’s medicine. It has been shown that high VEGF expression correlates with a bad prognosis in glioma patients (32). It has also been demonstrated that there is a relationship between the secretion of VEGF and irradiation since this therapeutic intervention leads to enhanced secretion of VEGF in combination with activation of VEGF signaling pathways (16). Furthermore, high VEGF levels seem to act as a survival factor for irradiated cancer cells, including glioma (30, 33, 34). This dilemma of therapeutic irradiation is accompanied by increased angiogenesis which is associated with higher VEGF levels after irradiation. Therefore, clinical and experimental trials suggest the combination of radiation and antiangiogenic therapy to overcome this problem (35–37); however, highly innovative and effective approaches are still missing.

In this study, we could underline stimulating effects of VEGF and also of irradiation on the motility of human GBM cell lines, whereas there were no similar effects on cell proliferation. On the other hand, the blockage of VEGF receptors by axitinib diminished VEGF and irradiation mediated effects. Moreover, these axitinib treated cells showed decreased proliferation and motility compared to controls. Additional VEGF did not enhance the effects of irradiation. We conclude that its impact is mainly dependent on autocrine and paracrine stimulation of the VEGF receptor by a rapid secretion of VEGF after irradiation, as has been proven in glioma (16).

### VEGF and Radiation Influence Cell Motility of GBM Cell Lines

Cellular motility plays an important role in the metastasis of GBM, which is characterized by fast relapses in the brain after surgery and other therapies. Our study points out the importance of VEGF in cellular motility. We observed stimulating effects by adding high concentrations of VEGF and comparable stimulating effects of irradiation in both GBM cell lines. Besides this we could reduce cellular motility in these cell lines using the VEGF receptor inhibitor axitinib, which points to VEGF being a key player in an autocrine stimulation of motility. Nevertheless, cellular motility was not completely impaired by axitinib, probably due to other signaling pathways that provoke migration, for instance through activation of the epidermal growth factor receptor (EGFR) pathways (38, 39). Our results match with those of Kil et al. (36), who showed an increased transwell migration of U-251 cells after VEGF stimulation or incubation in medium collected from GBM cells 72 h after irradiation. In that study, the stimulating effects of the irradiated medium could be blocked by VEGF antibodies, which were directly supplemented. These results in chemotactic experiments display the role of VEGF as a cytokine rather than its influence on the general motility of the cells. But a strong hint is given, that irradiation can stimulate the secretion of VEGF.

In the present study, we extend this previous knowledge to U-373 cells indicating a general mechanism at least in glioblastoma cells. We could show that VEGF is not only a chemoattractant, but also exerts stimulating effects on the general motility of these cells, too. Using videography as a technique to track living cells, we showed that there was an increased amount of undirected migration after VEGF exposure. However, our observations contradict the results of Ghosh et al. (40) who revealed a decrease in the migration of GBM cells after 2 Gy irradiation, but a strong increase at lower doses. This difference between the studies could be due to different radiation sources, since Ghosh et al. used a cobalt gamma radiation unit. Different dose rates and different photon energies might result in different responses from the cells to the same overall dose. However, we know from the data published by Hovinga et al. (41) and Kil et al. (36) that VEGF is a chemoattractant for GBM cells. Additionally, these groups also demonstrated that irradiation promotes secretion of VEGF in U-251 cells. In the present study, we detected a slight increase in VEGF levels in the supernatant of U-373 after irradiation, but not in U-251. It is likely that the amount of VEGF secretion in U-251 positively correlates with the dose of irradiation and the time point of VEGF measurement. Here, we used a single dose of 2 Gy irradiation, which reflects the dose that is common in fractionated radiotherapies of GBM (42), whereas other studies showing an increase in VEGF used different doses up to 20 Gy (36, 41). Besides this, we checked for VEGF levels 24 h after irradiation, the time point at which cell motility and cell proliferation was analyzed. The study by Kil in U-251 checked for VEGF levels 72 h after irradiation while the study by Hovinga et al. checked for VEGF 24, 48, and 72 h after irradiation with a minimum dose of 5 Gy. Nevertheless, it can be concluded, that in human GBM cell lines U-251 and U-373 VEGF and irradiation are able to speed up the cells, while axitinib has a strong decelerating effect. This is in line with *in vivo* data, in which high levels of VEGF are a negative factor for the prognosis (32). As the present data show that VEGF is a key player for migration, it is likely that forced invasion into the brain parenchyma is also driven by VEGF.

### VEGF Has an Autocrine Impact on Cell Proliferation

We also examined the effects of VEGF and irradiation on cell proliferation, for which VEGF acts as a stimulator in endothelial cells and astrocytes (7, 27). Tumor cells in general show high levels of activation in the mitogen-related pathways, for example activation of the EGFR, which acts as a potent mitogen (38, 39). But in contrast to other cell types, we were not able to detect any significant effects of irradiation or added VEGF on cell proliferation in U-251 and U-373 cells. However, 24 h of treatment with axitinib reduced proliferation significantly in both cell lines. Xu et al. (43) demonstrated an increase in the proliferation of enriched GBM stem cells after stimulation with VEGF (0.1 µg/ml). Although this is an interesting result, an enriched GBM stem cell culture is not directly comparable with the situation in GBM patients (44–46). In addition, *in vivo* studies showed a reduction in blood vessel infiltration of the tumor in line with a reduction in tumor size by means of anti-angiogenic treatment (47, 48). In 2012, Lee et al. (49) demonstrated that the reduction of tumor size in glioma xenografts after antiangiogenic drugs is not only caused by a reduction of angiogenic proliferation, but also by the dependency of the tumor cells on autocrine and paracrine VEGF stimulation. This autocrine dependency was also proved by Mesti et al. in GBM cells (2014). Moreover, this study showed that the VEGF antibody bevacizumab is not able to reduce proliferation, whereas SU1498, a VEGF-R2 blocker, had a distinct anti-proliferative impact after 72 h of incubation, but no effect after 24 h. It can be assumed that the lack of any stimulating VEGF effect on proliferation in our experiments is caused by a sufficient activation of VEGF signaling yet under control conditions. Additional proliferation experiments after 3 days of incubation in our lab yielded similar results than after 24 h, with no significant differences to 24 h values.

Up to now, little data are available with respect to effects of axitinib on glioma cells *in vitro* (50–52). In our study the decrease in cell proliferation through axitinib treatment points out the important role of autocrine VEGF receptor stimulation in U-251 and U-373 cells. This decrease is allegeable by the fact that axitinib leads to cell cycle arrest, recently shown in U-251 cells (53).

### VEGF Receptor Signaling in GBM

**Figure 4:**
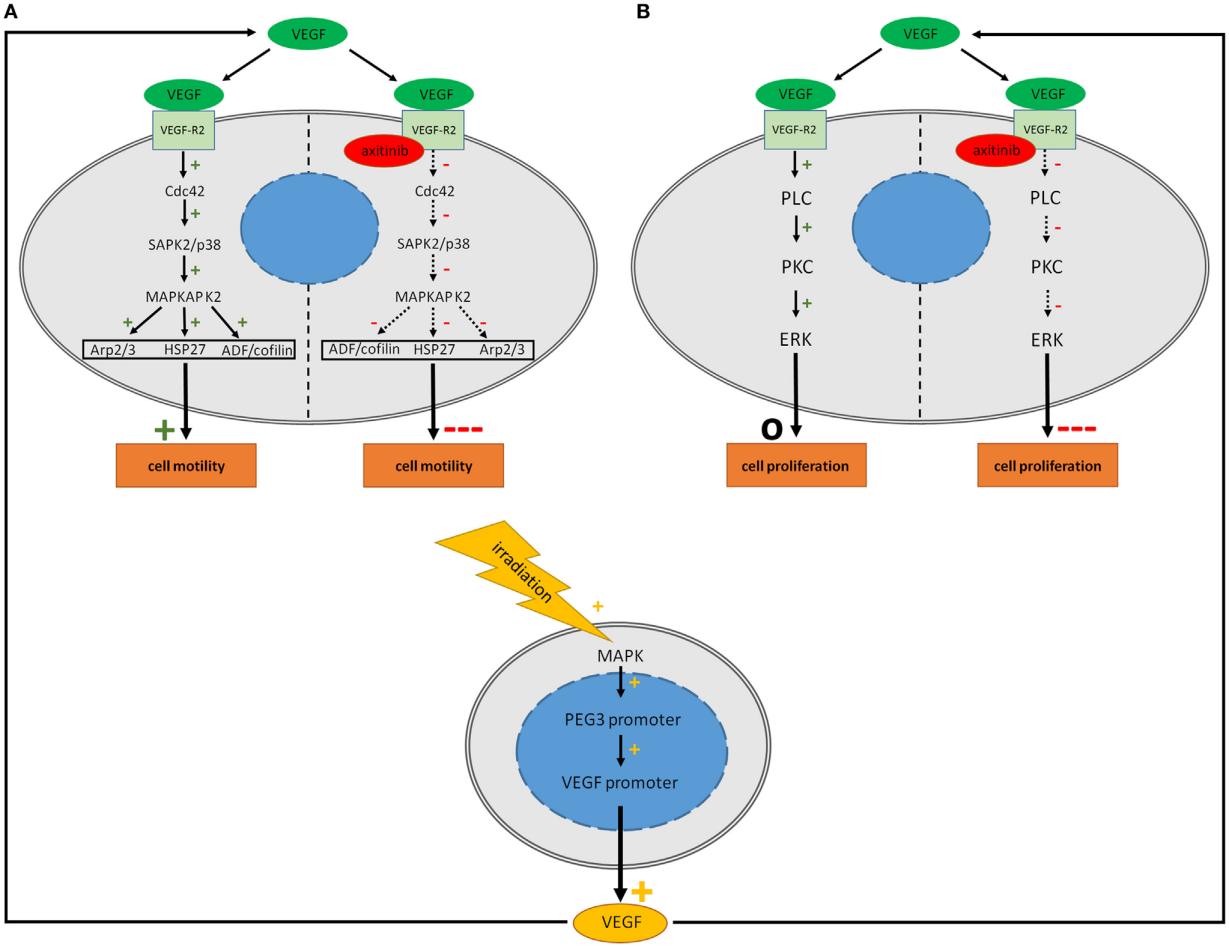
Potential signaling in human glioblastoma cell lines after treatment with vascular endothelial growth factor (VEGF), irradiation and axitinib. VEGF activates multiple pathways including the Cdc42 and PLC pathways concerning cell migration and proliferation. It is supposed that stimulating effects of irradiation are mediated *via* enhanced synthesis of VEGF. Irradiation elevates VEGF biosynthesis of glioblastoma multiforme cells *via* MAPK activation. **(A)** Activation of the Cdc42 pathway by VEGF leads to an increased activation of Arp 2/3, HSP27, and ADF/cofilin resulting in an enhanced motility. Blockade of the VEGF-R2 by axitinib might decrease the activation of the Cdc42 pathway due to theoretical consideration resulting in a crucial decreased cell motility. **(B)** VEGF activates the PLC pathway which is involved in cell proliferation while axitinib might deactivate this pathway with impairment of cell proliferation. While motility is increased by VEGF, no positive effect on proliferation could be observed. This might be due to fully activated VEGF-pathways even under control conditions. Continuous arrows: evidence of the effect, dotted arrows: assumption of the effect.

Vascular endothelial growth factor as a ligand for VEGF-R1 and VEGF-R2 activates several different pathways (Figures 4A,B). We demonstrated the expression of VEGF-R2 by immunohistochemistry and PCR in U-251 and U-373 cells. These data are in line with other studies that used different techniques such as Western blot, FACS and RT-PCR in different GBM cells (26, 30, 31, 54–57).

In glial cells, pathways which are activated by VEGF are less well understood, especially those that are linked to the actin network. Most VEGF dependent effects are mediated by VEGF-R2 (7). One very important aspect in the VEGF-R2 activation is the phosphorylation of tyrosine residue 1214. In endothelial cells, it was demonstrated that its phosphorylation, in combination with Cdc42 activation, leads to an activation of the SAPK2/p38 pathway, which stimulates MAPKAP K2 followed by phosphorylation of Hsp27 (Figure 4A). Furthermore, a stimulation of ADF/cofilin, Arp2/3, and WASP is involved in that pathway and acts as a re-organizer of the actin cytoskeleton with increased actin dynamics (13, 58, 59). Besides this, the focal adhesion kinase (FAK) seems to be an important factor in cell migration and invasion, as VEGF-R2 activation results in a phosphorylation of FAK in endothelial cells (60) and in glioma (36). FAK is a protein tyrosine kinase which is expressed in most tissues, with particularly high levels in the brain (61, 62). Treatment with cerivastatin reversibly blocks FAK phosphorylation and could be a new strategy to inhibit cell invasion (63). Another pathway of motility that is closely related to FAK is the tyrosine kinase src, which is phosphorylated by stimulation through VEGF (64). These effects are mediated by VEGF-R1 and -R2, but preferentially by VEGF-R2 signaling (36, 65, 66). In our study using PCR we showed semi-quantitatively that expression of VEGF-R1 (*FLT1*) is much lower than the expression of VEGF-R2 (*KDR*) which may explain that VEGF mediated effects in GBM are mainly based on VEGF-R2 signaling. Proliferation stimulating effects of VEGF-R2 are mediated by activation of phospholipase C following protein kinase C and extracellular signal regulated kinase (ERK) signaling (Figure 4B) (67, 68). All in all, these possible pathways might explain the role of VEGF signaling in cell motility and proliferation in GBM.

### The Role of VEGF and Irradiation in GBM

In conclusion, the effects of VEGF on GBM cells concerning motility are similar to the effects observed in astrocytes; however, in GBM cells the effects are slightly alleviated. One reason for this could be the high intrinsic VEGF production in GBM cells along with a relatively weak receptor expression level (31). We did not detect any stimulating effects of VEGF treatment on proliferation, which is not surprising, as there are other growth factors that might be over-stimulated in GBM. Even the concentration of endogenous VEGF under control conditions might be sufficient for a full activation of proliferation. However, in case that the VEGF receptor blocker axitinib passes through clinical trials, it could be a promising therapy for GBM patients, since two of the main characteristics of GBM could be disarmed; U-251 and U-373 lost their velocity, and proliferation could be reduced. Furthermore, irradiation mediated accelerating effects could be diminished. Therefore, a combination of axitinib and irradiation could be a potent strategy in the treatment of GBM.

## Ethics Statement

This article does not contain any studies with human participants or animals performed by any of the authors.

## Author Contributions

RK, VM, VT, HB, and CT conducted the study and analysis. RK, HB, IA, and CT contributed to the concept and design of this study. The paper was written by all authors. All authors approved the final article.

## Conflict of Interest Statement

All the authors declare that research has been conducted without any commercial or financial relationships which could be construed as a potential conflict of interest.

## Abbreviations

GBM, glioblastoma multiforme; VEGF, vascular endothelial growth factor; VEGF-R1, VEGF receptor 1; VEGF-R2, VEGF receptor 2.
